# Crucial Optimization of Translational Components towards Efficient Incorporation of Unnatural Amino Acids into Proteins in Mammalian Cells

**DOI:** 10.1371/journal.pone.0067333

**Published:** 2013-07-12

**Authors:** Liang Xiang, Kathryn Moncivais, Faqin Jiang, Blake Willams, Lital Alfonta, Zhiwen J. Zhang

**Affiliations:** 1 Department of Bioengineering, School of Engineering, Santa Clara University, Santa Clara, California, United States of America; 2 Biomedical Engineering Department, University of Texas at Austin, Austin, Texas, United States of America; 3 School of Pharmacy, Shanghai Jiaotong University, Shanghai, China; 4 Avram and Stella Goldstein-Goren Department of Biotechnology Engineering, The Ilse Katz Institute for Nanoscale Science and Technology, Ben-Gurion University of the Negev, Beer-Sheva, Israel; The Scripps Research Institute, United States of America

## Abstract

The ability to site-specifically incorporate unnatural amino acids (UAAs) into proteins is a powerful tool in protein engineering. While dozens of UAAs have been successfully introduced into proteins expressed by *Escherichia coli* cells, it has been much more challenging to create tRNA and tRNA-Synthetase pairs that enable UAAs incorporation, for use in mammalian systems. By altering the orthogonality properties of existing unnatural pairs, previously evolved pairs for use in *E. coli* could be used in mammalian cells. This would bypass the cumbersome step of having to evolve mutant synthetases and would allow for the rapid development of new mammalian pairs. A major limitation to the amount of UAA-containing proteins that can be expressed in the cell is the availability of UAA-charged orthogonal suppressor tRNA. By using a natural mammalian tRNA promoter, the amount of functional suppressor tRNA can be greatly increased. Furthermore, increasing recognition of the suppressor tRNA by the mutant synthetase will ultimately lead to the appearance of more UAA-charged tRNA.

## Introduction

Transfer ribonucleic acids (tRNAs) serve as an adaptor molecule, bridging between genetic information and amino acid sequences during protein biosynthesis. Aminoacyl-tRNA synthetases (aaRSs) catalyze the covalent attachment of amino acids to a corresponding tRNA and have evolved active sites that are highly specific for a given substrate [1]. Accuracy is of an utmost importance in such interactions since an incorrectly aminoacylated tRNA will still be used by the ribosome complex to extend the growing peptide chain so long as the anticodon-codon base pairing is correct. Many unnatural amino acids (UAAs) have been genetically incorporated into proteins by taking advantage of this phenomenon [2]. Several groups have used mutants of the *Methanocaldococcus jannaschii* (*M. jannaschii)* tyrosyl-tRNA synthetase (TyrRS) to charge an amber codon-suppressing tRNA with an UAA [3]. Once this pair has been introduced into an orthogonal host, the unnatural aaRS will charge the suppressor tRNA, thus allowing that cell to decode an UAA in response to the amber stop codon (TAG). As such, UAAs have been incorporated into proteins in several different applications, including small molecule labeling [4], structural studies of neuronal channels [5], site-specific cross-linking [6] and even electrochemical wiring of enzymes in biofuel cells [7].

All of the existing unnatural aaRSs derived from *M. jannaschii* TyrRS can only be used in prokaryotic cells; they are not orthogonal in eukaryotic systems [8], where they would charge endogenous tRNAs with the UAA. To the best of our knowledge, only a few unnatural aaRSs are available for incorporating UAAs in eukaryotic cells [9], as compared to the several dozens that can be used in prokaryotic cells. Evidence suggests that those domains on the TyrRS responsible for recognizing the amino acid and tRNA targets can be modified independently of one another [10], [11]. The existing set of unnatural aaRS derived from *M. jannaschii* TyrRS can thus be used in a eukaryotic system if they are manipulated into ignoring endogenous tRNAs.

We have previously shown that *M. jannaschii* TyrRS can be made orthogonal in mammalian cells by using peptide transplantation involving the connective polypeptide 1 (CP1) domain of *E. coli* TyrRS [11]. *M. jannaschii* that is not orthogonal in mammalian cells can be made orthogonal by switching C1:G72 to G1:C72 thus generating 1bp-tRNA_CUA_ [11]. The newly designed mutant TyrRS can then be paired with the engineered orthogonal amber codon-suppressing tRNA to form an orthogonal pair in mammalian cells capable of introducing a tyrosine to a protein in response to amber codon TAG. We reasoned that a similar CP1 transplantation could also be applied to an evolved *M. jannaschii* TyrRS (aaRS) specific to an unnatural amino acid in *E. coli* allowing it to charge 1bp-tRNA_CUA_ with the same unnatural amino acid in mammalian cells [11]. However, low suppression efficiency limited our ability to manipulate an unnatural aaRS in this manner. Herein, we describe our approaches to improve amber suppression efficiency in mammalian cells by increasing the expression of amino acid-charging suppressor tRNA and enhancing interactions between CP1-swapped TyrRS and its corresponding orthogonal amber codon-suppressing tRNA. This improved methodology can be applied in a ‘cut-and-paste’ manner to shuttle previously evolved unnatural aaRSs for use in mammalian systems.

## Results and Discussion

### High expression of functional archaeal tRNA in mammalian cells

**Figure 1:**
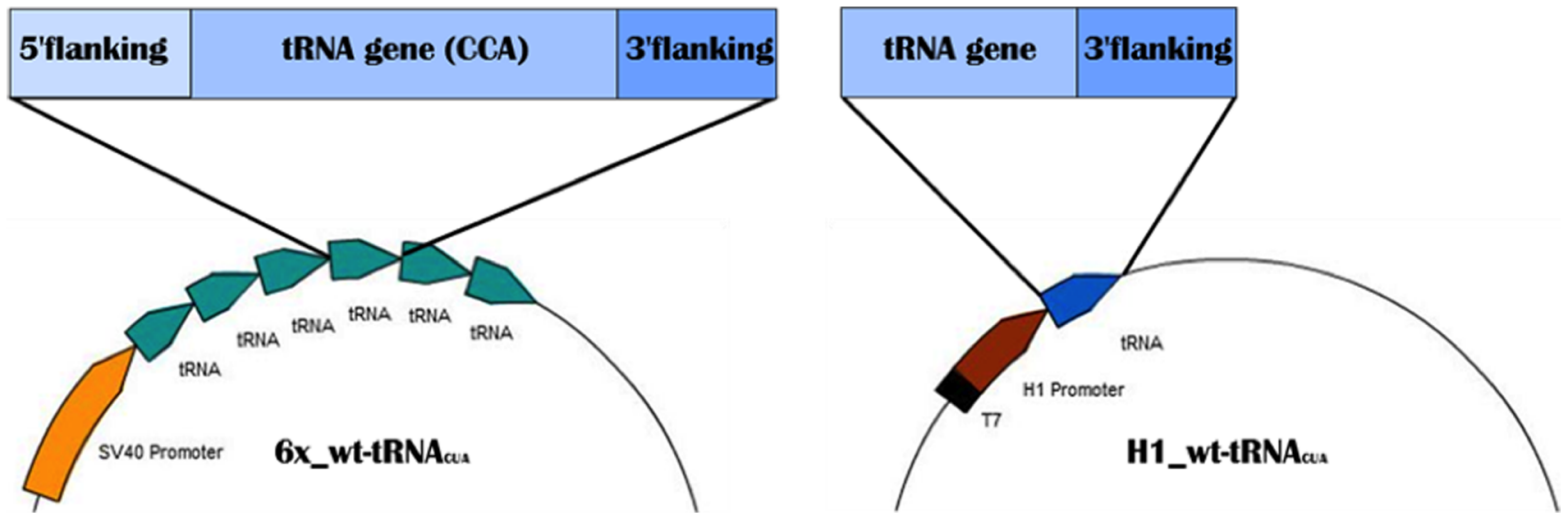
Comparison of the tandem copy and H1 promoter plasmid designs. The six-tandem approach (**left**) was replaced with a plasmid containing a human H1 promoter (**right**). A 3′ flanking sequence was also used to terminate transcription. The tRNA gene sequences are identical between the two plasmid designs, except in the 3′ CCA tail. The construct on the left contains this sequence while that on the right does not (EZ Plasmid Map).

In order to express archaebacterial *M. jannaschii* in mammalian cells, the tRNA was previously flanked with sequences taken from a human tyrosyl-tRNA gene. Six tandem repeats of the resulting genes were cloned into plasmid pZeoSV2 (+) to create 6x_wt-tRNA_CUA_ (**Figure 1**) [11]. However, this approach afforded low expression of the tRNA in mammalian cells due to the differences in the manner in which archaeal and eukaryotic cells initiate transcription of their tRNA genes and subsequently process the transcript [12]. Previously, the human H1 promoter [13] was used to over-express a downstream *E. coli* tyrosyl-tRNA gene in mammalian cells [5]. The concept was adopted here to over-express archeabacterial tRNA in mammalian cells. A human H1 promoter was inserted in front of an amber codon-suppressing archaebacterial tyrosyl-tRNA (3′-CCA removed). A single copy of the resulting gene was further cloned into plasmid pTRE-TIGHT-BI (Clontech) to afford a new vector H1_wt-tRNA_CUA_ (**Figure 1**).

**Figure 2:**
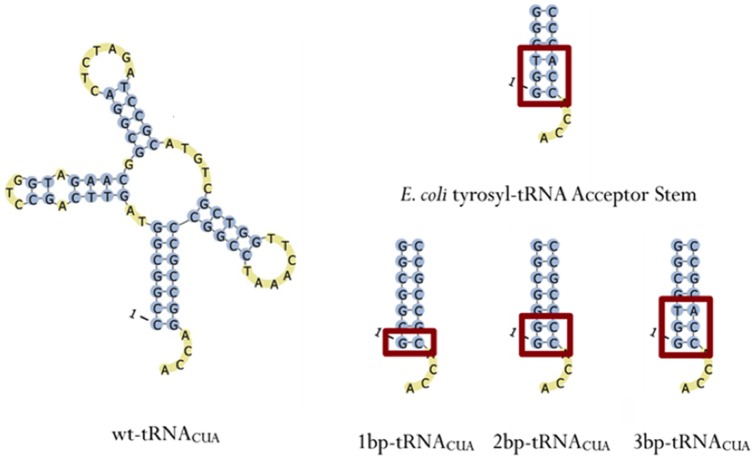
Acceptor stem sequences of the various tRNA constructs. Each tRNA construct was cloned into a plasmid, downstream of the human H1 promoter. Wt-tRNA_CUA_ is identical to *M. jannaschii* tyrosyl-tRNA, except in the anticodon region, where it contains a C35 (pknotsRG, BiBiServ).

**Figure 3:**
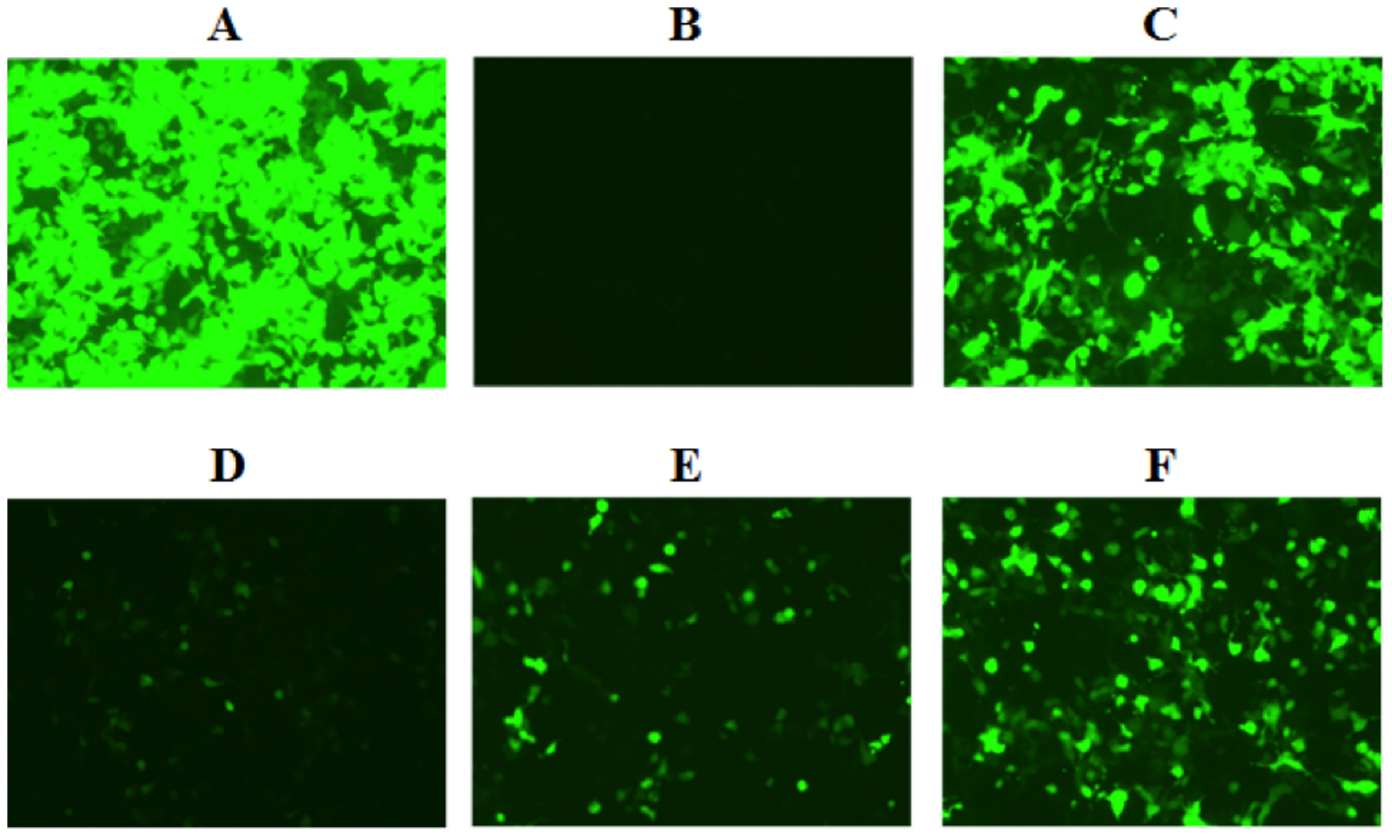
Assessing the orthogonality of various tRNA constructs in HEK293T cells. Full-length GFP was visualized by UV-light 72 hours after transfection. Expression of full-length GFP will only occur in the presence of amino acid charged suppressor tRNA. Since no exogenous aaRS was provided, full-length GFP expression implies that endogenous aaRS was able to recognize and charge the tRNA with an amino acid. Cells were transfected with the following plasmids: (A) p-EGFP-N1 (B) p-GFP_39TAG (C) p-GFP_39TAG and H1_wt-tRNA_CUA_ (D) p-GFP_39TAG and H1_1bp-tRNA_CUA_ (E) p-GFP_39TAG and H1_2bp-tRNA_CUA_ (F) p-GFP_39TAG and H1_3bp-tRNA_CUA_.

Studies have shown that the sequence of the 1:72 base pair within the acceptor stem of tyrosyl-tRNA is key in determining whether that tRNA can be recognized and charged by TyrRSs from other species [10]. Bacterial tyrosyl-tRNAs contain a G1:C72 base pair, while archaeal and eukaryotic tyrosyl-tRNAs contain a C1:G72 pair [8]. Sequences in the acceptor stem, in addition to the first base pair, have also been demonstrated to be important identity elements [14]. Thus, in order to identify the sequence in the acceptor stem that can be best recognized by *E. coli* CP1 region, three different *M. jannaschii* tyrosyl-tRNA genes were created, in which the first, second, and third base pairs of the acceptor stem were mutated to match those of the *E. coli* tyrosyl-tRNA (**Figure 2**). Each construct was cloned into a plasmid based on the parent vector H1_wt-tRNA_CUA_ to yield plasmids H1_1bp-tRNA_CUA_, H1_2bp-tRNA_CUA_, and H1_3bp-tRNA_CUA_, respectively. A functional suppression assay was then employed to test the orthogonality of these amber-suppressor tRNAs in mammalian cells. In this assay, an amber stop codon TAG replaced the codon for the 39th amino acid (tyrosine) of green fluorescent protein (GFP) gene in vector p-EGFP-N1 to afford p-GFP_39TAG. Mammalian HEK 293T cells were co-transfected with p-GFP_39TAG and the plasmid expressing the amber-suppressor tRNA. Suppression of the 39th amber codon TAG led to the expression of a full-length GFP *in vivo* (**Figure 3**), and fluorescence-activated cell sorting (FACS) was used to quantify the number of fluorescent cells 72 hours after transfection (**Table 1**). The low level of detectable GFP signal indicates the tRNA is not charged by endogenous aaRSs and is therefore orthogonal to the mammalian cell's translational machinery.

**Table 1:** Assessing the orthogonality of various tRNA constructs in HEK293T cells.

Plasmids used		Fluorescent Cells ± STD
p-EGFP-N1	-	97.4% ± 1.07%
p-GFP_39TAG	-	0.2% ± 0.01%
p-GFP_39TAG	H1_wt-tRNACUA	32.4% ± 0.91%
p-GFP_39TAG	H1_1bp-tRNACUA	1.7% ± 0.16%
p-GFP_39TAG	H1_2bp-tRNACUA	5.9% ± 0.70%
p-GFP_39TAG	H1_3bp-tRNACUA	9.6% ± 0.15%

72 hours after transfection, FACS was used to measure the population of fluorescent cells.

FACS analysis showed that introduction of TAG into the GFP gene resulted in the generation of almost no detectable full-length GFP (**Table 1**). Wt-tRNA_CUA_ does not contain any acceptor stem modifications. In spite of this, when expressed in HEK 293T cells, a ∼33% suppression of the amber stop codon was observed, indicating Wt-tRNA_CUA_ can be charged by endogenous mammalian aaRSs and is not orthogonal in mammalian cells [8]. The high suppression efficiency of this non-orthogonal tRNA shows that the human H1 promoter can be used to drive the expression of large amounts of a mature and functional suppressor tRNA from an archaeally-derived gene.

We then mutated the first base pair in the tRNAs acceptor stem from C1:G72 to G1:C72, yielding 1bp-tRNA_CUA_. In this case, an exogenous aaRS would be necessary to charge 1bp-tRNA_CUA_ with an amino acid. After it is aminoacylated, this tRNA would be able to incorporate its attached amino acid into a protein in response to a TAG codon. We were surprised to find that additional mutations in the second and third base pairs of the acceptor stem resulted in a tRNA that was less orthogonal (**Table 1**).

### Engineering of M. jannaschii TyrRS with specificity for 1bp-tRNA_CUA_

**Figure 4:**
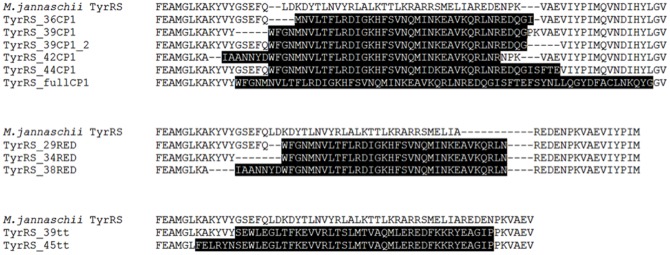
Alignment of the amino acid sequences of the CP1-transplanted mutants. Inserted CP1 sequences are shown in black. Six sequences used were from *E. coli* TyrRS, while two were taken from *T. thermophilus* TyrRS. Both bacterial TyrRSs recognize a G1:C72 containing tyrosyl-tRNA. Each CP1 swapped TyrRS was tested for the ability to charge 1bp-tRNA_CUA_ in HEK293T cells. (Bioworkbench, SDSC).

Our next focus was manipulating the *M. jannaschii* TyrRS into recognizing the G1:C72-containing 1bp-tRNA_CUA_ efficiently. This method should also be applicable for an unnatural aaRS that would allow 1bp-tRNA_CUA_ to be charged with an UAA. Previous work demonstrated the importance of the TyrRS connective peptide 1 (CP1) region in recognizing its corresponding tRNA acceptor stem [10], [11]. We constructed eleven different variants of *M. jannaschii* TyrRSs (**Figure 4**) containing CP1 sequences taken from bacterial TyrRSs that recognize G1:C72-containing tyrosyl-tRNAs. All designs were tested by using the previously described functional suppression assay (**Table 2**). Alignment of the crystal structures of the *E. coli* (pdb.org 1X8X) and *M. jannaschii* TyrRS (pdb.org 1J1U) were used to design six *E. coli* CP1 swapped mutants: TyrRS_36CP1, TyrRS_39CP1, TyrRS_39CP1_2, TyrRS_42CP1, TyrRS_44CP1, and TyrRS_fullCP1. Amino acid sequence alignment (CLUSTAW, Bioworkbench, SDSC) of the two TyrRS was used to design another three CP1-transplanted mutants: TyrRS_29RED, TyrRS_34RED, and TyrRS_38RED. The N-terminal regions of these swaps are similar to the structural homology designs, although the C-terminal region is based on a RED sequence that is shared by both TyrRSs. The crystal structure of *Thermus thermophilus* (*T. thermophilus*) TyrRS is also available, and it is known to charge a G1:C72-containing tyrosyl-tRNA [15]. Based on the structural alignment between *T. thermophilus* TyrRS and the *M. jannaschii* TyrRS, two additional CP1-substituted mutants were designed: TyrRS_39tt and TyrRS_45tt. All CP1-swapped TyrRSs also contained an Arg286 mutation that has previously been shown to increase recognition of the anticodon region of a suppressor tRNA by *M. jannaschii* TyrRSs [16].

**Table 2:** Ratio of the suppression efficiency to tRNA background.

	Suppression Efficiency	Ratio
tRNA background	1.70%	1
*E. coli* TyrRS	22.02%	13.0
*M. jannaschii* TyrRS	3.96%	2.3
TyrRS_36CP1	1.83%	1.1
TyrRS_39CP1	2.18%	1.3
TyrRS_39CP1_2	6.58%	3.9
TyrRS_42CP1	5.06%	3.0
TyrRS_44CP1	9.41%	5.5
TyrRS_fullCP1	1.98%	1.2
TyrRS_29RED	2.14%	1.3
TyrRS_34RED	2.84%	1.7
TyrRS_38RED	3.33%	2.0
TyrRS_39tt	3.11%	1.8
TyrRS_45tt	3.66%	2.2

tRNA background reflects the suppression efficiency of 1bp-tRNA_CUA_ when no exogenous aaRS was provided. Both TyrRS_44CP1 and TyrRS_39CP1_2 recognized 1bp-tRNA_CUA_ well and were able to charge it in HEK293T cells.

HEK293T cells were transfected with p-GFP_39TAG, H1_1bp-tRNA_CUA_, and with a plasmid containing a gene for a CP1-swapped TyrRS, harvested 72 hours later, and analyzed by FACS analysis. Since the tRNA is not recognized by any endogenous aaRS, full-length GFP could only be detected when the 1bp-tRNA_CUA_ was charged by the CP1-swapped TyrRS. Wild type *E. coli* TyrRS was used as a positive control and offered a 13.5-fold increase in fluorescence over that seen when cells were transfected with a plasmid harboring 1bp-tRNA_CUA_ only (used as background, **Table 2**). The high suppression efficiency of this pair (∼23%) demonstrated that 1bp-tRNA_CUA_ is a functional amber codon-suppressing tRNA as long as it can be charged with an amino acid. Wild type *M. jannaschii* TyrRS also recognized the 1bp-tRNA_CUA_ to some degree and led to a doubling in the suppression efficiency, when compared to the background. TyrRS_36CP1, TyrRS_29RED, and TyrRS_fullCP1 all failed to recognize 1bp-tRNA_CUA_ and did not yield any significant increase in fluorescence. We were surprised that TyrRS_fullCP1 performed so poorly since a full-length CP1 swap between human TyrRS and bacterial TyrRS was previously shown to be functional [10]. Neither of the *T. thermophilus* CP1 transplantations was able to recognize 1bp-tRNA_CUA_ very well. The most active CP1-swapped mutant was TyrRS_44CP1, although TyrRS_39CP1_2 also produced an almost four-fold increase in interaction over the background level.

The optimized pair composed of TyrRS_44CP1 and H1_1bp-tRNA_CUA_ achieved a suppression efficiency of > 9%, as compared to the <2% efficiency achieved with our previous pair [11]. Our next step will be to apply the CP1 transplantation method to an unnatural aaRS that was evolved from *M. jannaschii* TyrRS. This CP1-swapped unnatural aaRS can be combined with 1bp-tRNA_CUA_ in a mammalian system to allow the host to genetically encode an UAA in response to TAG. A five-fold increase in suppression efficiency should allow us to produce more unnatural amino acid-incorporated protein when using an unnatural aaRS.

In conclusion, we were able to show that by rationally designing the two crucial components, tRNA and a corresponding aaRS, we can improve: a) orthogonality in the mammalian host system; b) stop codon-suppression efficiency; c) expression of a protein, reaching yields up to five-fold higher than realized with previously used systems that rely on the amber suppression approach in mammalian cells. These improvements pave the way for efficient and specific incorporation of UAA into recombinant mammalian proteins.

## Materials and Methods

### Cell Cultures and Transfections

All plasmids were amplified in *E. coli* TOP10 cells and isolated using a Qiagen Miniprep Kit. HEK293T cells (ATCC^®^ CRL-11268^TM^) were cultured in Dulbecco's Modified Eagle Medium (D-MEM) supplemented with 10% (v/v) fetal bovine serum (Atlanta Biologicals, #C0136) in a 37°C humidified incubator containing 5% CO_2_. Transfections were performed using FuGENE HD according to product manual (Roche, Indianapolis, IN). Full-length GFP was excited at 460-500 nm and detected using a Nikon Eclipse TE2000-S microscope equipped with a FITC HyQ filter (Chroma).

### Construction of the p-GFP_39TAG Plasmid

The commercially available plasmid p-EGFP-N1 (Clontech) was used to express a gene for EGFP using a CMV promoter. The 39th codon in the EGFP gene was mutated from TAC to TAG by site-directed mutagenesis (SDM) using primers designed by the Stratagene online primer design program

5′-GCGAGGGCGAGGGCGATTAGACCTACGGCAAGC-3′

5′-GCTTGCCGTAGGTCTAATCGCCCTCGCCCTCGC-3′

A QuikChange II Site-Directed Mutagenesis Kit was purchased from Stratagene (Stratagene, Agilent Technologies, Santa Clara, CA), and site-directed mutagenesis was completed according to the provided protocol. Plasmids were sequenced by the University of Texas at Austin ICMB DNA Sequencing Facility. The new plasmid created was termed plasmid GFP_39TAG.

### Construction of the Human H1 Promoter tRNA-incorporating Plasmid (H1_wt-tRNA_CUA_)

The MCS-1 region from a commercially available plasmid, pTRE-TIGHT-BI (Clontech) was removed by digesting the plasmid with the KpnI and EcoRI restriction enzymes (NEB, Ipswich, MA). Oligonucleotides, containing a sequence for the T7 promoter:

5′-GGGGTACCCCTAATACGACTCACTATAGGGGGAATTCC-3′

5′-GGAATT-CCCCCTATAGTGAGTCGTATTAGGGGTACCCC-3′

were annealed and digested with KpnI and EcoRI. The digested insert was then ligated using DNA T4 Ligase into the pre-digested p-TRE-TIGHT-BI plasmid to form plasmid p-TRE-TIGHT-T7. Next, oligonucleotides containing the sequence for the human H1 promoter were annealed and extended using a Klenow enzyme. The oligonucleotide sequences were:

5′-CAACCCGCTCCAAGGAATCGCGGGCCCAGTGTCACTAGGCGGGAAC ACCCAGCGCGCGTGCGCCCTGGCAGGAAGATGGCTGTGAGGGACAGGGGAGTGGCGCCCTGCAA-3′

5′-GAACTTATAAGATTCCCAAATCCAAAGACATTTCACGTTTATGGTG ATTTCCCAGAACACATAGCGACATGCAAATATTGCAGGGCGCCACTCCCCTGTCCCTCACAGCC-3′

The sequence was then amplified by PCR using the primers:

5′-GGAATTCCAATTCGAACGCTGACGTCATCAACCCGCTCCAAGG AATC-3′

5′-GAAGATCTGTGGTCTCATACAGAACTTATAAGATTCCCA-3′

The resulting product was digested with the KpnI and BglII restriction enzymes and ligated into a pre-digested p-TRE-TIGHT-T7 plasmid to generate plasmid pT7-H1.

### Construction of the CP1-Swapped TyrRS Plasmids

TyrRS_44CP1 was created by replacing the CP1 region of *M. jannaschii* TyrRS (amino acids 110-148) with the CP1 region of *E. coli* TyrRS (amino acids 129-172). The 5′ end of the gene (containing sequences for amino acids 1-109) was amplified using the following primers:

5′-AAGGATCCACCATGGACGAATTTGAAATGAT-3′

5′-ACATTCATATTGCCGAACCACTGGAATTCACTTCCAT-3′

The 3′ end (containing amino acids 149-306) was amplified by PCR using primers:

5′-AGGGGATTTCGTTCACTGAGGTTATCTATCCAATAATGCA-3′

5′-CCCGAATTCTAATCTCTTTCTAATTGGCT-3′

Finally, the *E. coli* CP1 region (amino acids 129-172) was amplified from a wild type *E. coli* TyrRS gene by PCR using the following primers:

5′-ATGGAAGTGAATTCCAGTGGTTCGGCAATATGAATGT-3′

5′-TGCATTATTGGATAGATAACTTCAGTGAACGAAATCCCCT-3′

All three PCR fragments were combined, denatured for 15 minutes at 85°C and elongated with a Klenow enzyme for 30 minutes at room temperature. The Klenow product was again amplified by PCR, purified, and digested with the HindIII and EcoRI restriction enzymes. Finally, TyrRS_44CP1 was created by ligating the digested product into a pre-digested pEF6-V5-His6-TOPO plasmid (Invitrogen, Carlsbad, CA) using T4 DNA ligase (NEB). All other CP1-transplanted mutants were created by PCR in a process similar to that described in the previous section. The chimeric gene was divided into three segments, and each was amplified by PCR. Finally, a joint gene was also amplified by PCR, digested, and ligated into plasmid pEF6-V5-His6-TOPO. Primers are listed for each of the segments, based on the size and sequence of the CP1 swap.

TyrRS_36CP1:

5′-AAGGATCCACCATGGACGAATTTGAAATGAT-3′

5′-CGCAGGAAGGTCAGCACATTCATCTGGAATTCACTTCCATAAACAT-3′

5′-ATGTTTATGGAAGTGAATTCCAGATGAATGTGCTGACCTTCCTGCG-3′

5′-TGGATAGATAACTTCAGCAACAATCCCCTGATCTTCACGGTTGAGA-3′

5′-TCTCAACCGTGAAGATCAGGGGATTGTTGCTGAAGTTATCTATCCA-3′

5′-TGCATTATTGGATAGATAACTTCAGTGAACGAAATCCCCT-3′

TyrRS_39CP1:

5′-AAGGATCCACCATGGACGAATTTGAAATGAT-3′

5′-ACATTCATATTGCCGAACCAATAAACATATTTTGCCTT-3′

5′-AGGCAAAATATGTTTATTGGTTCGGCAATATGAATGT-3′

5′-ATAACTTCAGCAACCTTTGGCCCCTGATCTTCACGGTTGA-3′

5′-TCAACCGTGAAGATCAGGGGCCAAAGGTTGCTGAAGTTAT-3′

5′-TGCATTATTGGATAGATAACTTCAGTGAACGAAATCCCCT-3′

TyrRS_39CP1_2:

5′-GCGGATCCGCCACCATGGACGAATTTGAAATGATAAAGAGAA-3′

5′-ACATTCATATTGCCGAACCAATAAACATATTTTGCCTT-3′

5′-AAGGCAAAATATGTTTATTGGTTCGGCAATATGAATGT-3′

5′-ATAACTTCAGCAACCTTTGGCCCCTGATCTTCACGGTTGA-3′

5′-TCAACCGTGAAGATCAGGGGCCAAAGGTTGCTGAAGTTAT-3′

5′-GCTCTAGAGCTTATAATCTCTTTCTAATTGGCTCTAAAATCTTTATA AGTTCTTCAGCTACAGCATTTTTTAACCTCATTGGATGCAATTCCTT-3′

TyrRS_42CP1:

5′-GCGGATCCGCCACCATGGACGAATTTGAAATGATAAAGAGAA-3′

5′-TCATAGTTGTTCGCCGCGATTGCCTTTAACCCCATTGCTT-3′

5′-AAGCAATGGGGTTAAAGGCAATCGCGGCGAACAACTATGA-3′

5′-TTCAGCAACCTTTGGATTACGGTTGAGACGCTGCTTAACC-3′

5′-GGTTAAGCAGCGTCTCAACCGTAATCCAAAGGTTGCTGAA-3′

5′-GCTCTAGAGCTTATAATCTCTTTCTAATTGGCTCTAAAATCTTTATA AGTTCTTCAGCTACAGCATTTTTTAACCTCATTGGATGCAATTCCTT-3′

TyrRS_44CP1:

5′-AAGGATCCACCATGGACGAATTTGAAATGAT-3′

5′-ACATTCATATTGCCGAACCACTGGAATTCACTTCCAT-3′

5′-AGGGGATTTCGTTCACTGAGGTTATCTATCCAATAATGCA-3′

5′-CCCGAATTCTAATCTCTTTCTAATTGGCT-3′

5′-ATGGAAGTGAATTCCAGTGGTTCGGCAATATGAATGT-3′

5′-TGCATTATTGGATAGATAACTTCAGTGAACGAAATCCCCT-3′

TyrRS_fullCP1:

5′-AAGGATCCACCATGGACGAATTTGAAATGAT-3′

5′-ACATTCATATTGCCGAACCAATAAACATATTTTGCCTT-3′

5′-AGGCAAAATATGTTTATTGGTTCGGCAATATGAATGT-3′

5′-CCAACTGCAACATCAACGCCACCGTACTGTTTGTTCAGAC-3′

5′-GTCTGAACAAACAGTACGGTGGCGTTGATGTTGCAGTTGG-3′

5′-TGCATTATTGGATAGATAACTTCAGTGAACGAAATCCCCT-3′

TyrRS_29RED:

5′-GCGGATCCGCCACCATGGACGAATTTGAAATGATAAAGAGAA-3′

5′-ACATTCATATTGCCGAACCAATAAACATATTTTGCCTT-3′

5′-AAGGCAAAATATGTTTATTGGTTCGGCAATATGAATGT-3′

5′-TTGGATTTTCATCCTCTCTGTTGAGACGCTGCTTAACCGC-3′

5′-GCGGTTAAGCAGCGTCTCAACAGAGAGGATGAAAATCCAA-3′

5′-GCTCTAGAGCTTATAATCTCTTTCTAATTGGCTCTAAAATCTTTATA AGTTCTTCAGCTACAGCATTTTTTAACCTCATTGGATGCAATTCCTT-3′

TyrRS_34RED:

5′-AAGGATCCACCATGGACGAATTTGAAATGAT-3′

5′-ACATTCATATTGCCGAACCAATAAACATATTTTGCCTT-3′

5′-AGGCAAAATATGTTTATTGGTTCGGCAATATGAATGT-3′

5′-TTGGATTTTCATCCTCTCTGTTGAGACGCTGCTTAACCGC-3′

5′-GCGGTTAAGCAGCGTCTCAACAGAGAGGATGAAAATCCAA-3′

5′-TGCATTATTGGATAGATAACTTCAGTGAACGAAATCCCCT-3′

TyrRS_38RED:

5′-GCGGATCCGCCACCATGGACGAATTTGAAATGATAAAGAGAA-3′

5′-TCATAGTTGTTCGCCGCGATTGCCTTTAACCCCATTGCTT-3′

5′-AAGCAATGGGGTTAAAGGCAATCGCGGCGAACAACTATGA-3′

5′-TTGGATTTTCATCCTCTCTGTTGAGACGCTGCTTAACCGC-3′

5′-GCGGTTAAGCAGCGTCTCAACAGAGAGGATGAAAATCCAA-3′

5′-GCTCTAGAGCTTATAATCTCTTTCTAATTGGCTCTAAAATCTTTATA AGTTCTTCAGCTACAGCATTTTTTAACCTCATTGGATGCAATTCCTT-3′

TyrRS_39tt:

5′-AAGGATCCACCATGGACGAATTTGAAATGAT-3′

5′-GTGAGGCCCTCCAGCCACTCGGAATAAACATATTTTGCCTTTAAC-3′

5′-GTTAAAGGCAAAATATGTTTATTCCGAGTGGCTGGAGGGCCTCAC-3′

5′-ATAACTTCAGCAACCTTTGGGGGAATCCCCGCCTCGTACCGCTTC-3′

5′-GAAGCGGTACGAGGCGGGGATTCCCCCAAAGGTTGCTGAAGTTAT-3′

5′-TGCATTATTGGATAGATAACTTCAGTGAACGAAATCCCCT-3′

TyrRS_45tt:

5′-AAGGATCCACCATGGACGAATTTGAAATGAT-3′

5′-CTCGGAGTTGTAGCGGAGCTCAAATAACCCCATTGCTTCAAAAAC-3′

5′-GTTTTTGAAGCAATGGGGTTATTTGAGCTCCGCTACAACTCCGAG-3′

5′-ATAACTTCAGCAACCTTTGGGGGAATCCCCGCCTCGTACCGCTTC-3′

5′-GAAGCGGTACGAGGCGGGGATTCCCCCAAAGGTTGCTGAAGTTAT-3′

5′-TGCATTATTGGATAGATAACTTCAGTGAACGAAATCCCCT-3′.

### Construction of H1_1bp-tRNA_CUA_, H1_2bp-tRNA_CUA_, H1_3bp-tRNA_CUA_

Two oligonucleotides for the 1bp-tRNA_CUA_ gene were annealed, extended using a Klenow enzyme, and amplified by PCR. The sequences of oligonucleotides were:

5′-GAAGATCTGCGGCGGTAGTTCAGCCTGGTAGAACGGCGGACTCT AAATCCGCATGTCGCTGGTTCAAATCCGGCCCGCCGCAGACAAGTGCGGTTTTTTT-3

5′-CCAATGCATTGGTTGCCCGCTCGAGTAGAAAAAAACCGCACTTGTC TGCGGCGGGCCGGATTTGAACCAGCGACATGCGGATTTAGAGTCCGCCGTTCTA-3′

The PCR product was then digested with the BglII and PstI restriction enzymes and ligated into a pre-digested pT7-H1 (described above) plasmid to create the 1bp-tRNA_CUA_ plasmid.

H1_2bp-tRNA_CUA_ was created in a similar fashion using the following oligonucleotides to create the tRNA gene:

5′-GAAGATCTGGGGCGGTAGTTCAGCCTGGTAGAACGGCGGACTCTA AATCCGCATGTCGCTGGTTCAAATCCGGCCCGCCCCAGACAAGTGCGGTTTTTTT-3′

5′-CCAATGCATTGGTTGCCCGCTCGAGTAGAAAAAAACCGCACTTGTC TGGGGCGGGCCGGATTTGAACCAGCGACATGCGGATTTAGAGTCCGCCGTTCTA-3

Finally, H1_3bp-tRNA_CUA_ was generated using the following oligonucleotides:

5′-GAAGATCTGGTGCGGTAGTTCAGCCTGGTAGAACGGCGGACTCTA AATCCGCATGTCGCTGGTTCAAATCCGGCCCGCACCAGACAAGTGCGGTTTTTTT-3′

5′-CCAATGCATTGGTTGCCCGCTCGAGTAGAAAAAAACCGCACTTG TCTGGTGCGGGCCGGATTTGAACCAGCGACATGCGGATTTAGAGTCCGCCGTTCTA-3′.

### Constructing the E. coli TyrRS Plasmid

A gene for *E. coli* TyrRS was amplified from a plasmid by PCR using the following primers:

5′-GCTCTAGATTATTTCCAGCAAATCAGACAGTA-3′

5′-CGGGATCCATGGCAAGCAGTAACTTGATTAAA-3′

The PCR product was digested with the XbaI and BamHI restriction enzymes (New England Biolabs, Ipswich, MA) and ligated using T4 ligase into a pre-digested pEF6-V5 plasmid to express *E. coli* TyrRS in HEK293T cells.

All new sequences of TyrRSs have been deposited in GenBank with the following accession numbers:

TyrRS_36CP1: KF050826

TyrRS_39CP1: KF050827

TyrRS_39CP1_2: KF050828

TyrRS_42CP1: KF050829

TyrRS_44CP1: KF050830

TyrRS_fullCP1: KF050831

TyrRS_29RED: KF050832

TyrRS_34RED: KF050833

TyrRS_38RED: KF050834

TyrRS_39tt: KF050835

TyrRS_45tt: KF050836

### FACS Analysis

A BD FACSCalibur flow cytometer was used to gate approximately 10,000 cells, based on forward and side scatter. Each cell was then excited at a wavelength of 488 nm and the resulting emission was detected with an FL-1 filter (515–545 nm). Positive fluorescent populations were gated based on negative or positive controls and analyzed using Cyflogic v.1.2.1 computer software (CyFlo, Ltd., Turku, Finland).
